# Treatment of Hernias of Long Standing

**Published:** 1881-12

**Authors:** 


### Treatment of Hernias of Long Standing.

The following conclusions are reached by M. Thiry, in a paper on the above subject, presented by him to the Royal Belgian Academy of Medicine: (1.) Old hernias of large extent, constituting a variety of eventration, are susceptible of reduction in most cases. (2.) The large volume of a hernia is never a contra-indication to its reduction, although it necessitates the adoption of certain precautions, and the employment of considerable time. (3.) The diminution in the capacity of the abdominal cavity, in old hernias, is never antagonistic to a slow, methodical, and progressive taxis. (4.) By slowly re-entering the abdominal cavity, the extended parts gradually resume their former places. (5.) The best method of reduction is the "compressing taxis," which consists in only restoring organs to their natural position after they have been relieved, by pressure, of any vascular engorgement. Those organs which effected an exit last should be first replaced. (6.) In this variety of hernia, an elastic truss, adapted to the gradually decreasing size of the tumor, should be attached to an abdominal belt. (7.) When the hernia has been completely reduced, the projecting knob of the truss, with properly shaped convexity, should penetrate the ring and adapt itself to its dimensions.—*Bulletin de L'Academie Royale de Medecine de Belgique*, vol. xv., No. 6.

